# Directed induction of chondrogenic cells from mouse dermal fibroblast culture

**DOI:** 10.1186/ar3582

**Published:** 2012-02-09

**Authors:** Noriyuki Tsumaki

**Affiliations:** 1 Center for iPS Cell Research and Application, Kyoto University, Japan

## 

Repair of cartilage injury with hyaline cartilage has been a challenging clinical problem. Articular cartilage damage sometimes heals with fibrocartilage, which is different from hyaline cartilage. Fibrocartilage is a type of scar tissue that expresses types I and II collagen. In contrast, hyaline cartilage does not express type I collagen. When aiming to induce hyaline chondrogenic cells directly from dermal fibroblasts, in addition to activation of cartilage-specific matrix genes, elimination of expression of type I collagen is needed for generation of hyaline cartilage. Otherwise, the presence of type I collagen impairs cartilage extracellular matrix architecture, which leads to formation of fibrocartilage. The generation of induced pluripotent stem cells has provided a tool for reprogramming dermal fibroblasts to an undifferentiated state by ectopic expression of reprogramming factors. We found that retroviral expression of two reprogramming factors (c-Myc and Klf4) and one chondrogenic factor (SOX9) induces polygonal chondrogenic cells directly from adult dermal fibroblast cultures. Induced cells expressed marker genes for chondrocytes but not fibroblasts; the promoters of type I collagen genes were extensively methylated. Transduction of c-Myc, Klf4, and SOX9 produced two types of cells: chondrogenically reprogrammed cells and partially reprogrammed intermediate cells. Chondrogenically reprogrammed cells generated stable homogenous hyaline cartilage-like tissue without tumor formation when subcutaneously injected into nude mice. Hyaline cartilage-like tissue expressed type II collagen but not type I collagen. On the other hand, partially reprogrammed intermediate cells expressed type I collagen and produced tumor when injected into nude mice. Induced chondrogenic cells did not undergo pluripotent state during induction from dermal fibroblast culture, as time-lapse observation did not detect GFP reporter expression during induction from dermal fibroblasts prepared from transgenic mice in which GFP is inserted into the *Nanog *locus. These results suggest that chondrogenic cells induced by this approach are free from a risk of teratoma formation which associates with cells prepared through generation of iPS cells followed by redifferentiation into the target cell type. The dox-inducible induction system demonstrated that induced cells are able to respond to chondrogenic medium by expressing endogenous *Sox9 *and maintain chondrogenic potential after substantial reduction of transgene expression. This approach could lead to the preparation of hyaline cartilage directly from skin, without going through pluripotent stem cells, in future regenerative medicine.

